# A Parasitic Arsenic Cycle That Shuttles Energy from Phytoplankton to Heterotrophic Bacterioplankton

**DOI:** 10.1128/mBio.00246-19

**Published:** 2019-03-19

**Authors:** Stephen J. Giovannoni, Kimberly H. Halsey, Jimmy Saw, Omran Muslin, Christopher P. Suffridge, Jing Sun, Chih-Ping Lee, Eric R. Moore, Ben Temperton, Stephen E. Noell

**Affiliations:** a Department of Microbiology, Oregon State University, Corvallis, Oregon, USA; University of Oklahoma; Duquesne University; University of Hawai‘i at Mānoa

**Keywords:** SAR11, arsenic, ocean

## Abstract

In many regions of the world oceans, phytoplankton face the problem of discriminating between phosphate, an essential nutrient, and arsenate, a toxic analogue. Many phytoplankton, including the most abundant phytoplankton group known, *Prochlorococcus*, detoxify arsenate (AsV) by reduction to arsenite (AsIII), followed by methylation and excretion of the methylated arsenic products. We synthesized [^14^C]dimethyl arsenate (DMA) and used it to show that cultured *Pelagibacter* strain HTCC7211 (SAR11) cells oxidize the methyl group carbons of DMA, producing ^14^CO_2_ and ATP. We measured [^14^C]DMA oxidation rates in the P-depleted surface waters of the Sargasso Sea, a subtropical ocean gyre. [^14^C]DMA was oxidized to ^14^CO_2_ by Sargasso Sea plankton communities at a rate that would cause turnover of the estimated DMA standing stock every 8.1 days. SAR11 strain HTCC7211, which was isolated from the Sargasso Sea, has a pair of arsenate resistance genes and was resistant to arsenate, showing no growth inhibition at As/P ratios of >65:1. Across the global oceans, there was a strong inverse relationship between the frequency of the arsenate reductase (LMWPc_ArsC) in *Pelagibacter* genomes and phosphate concentrations. We propose that the demethylation of methylated arsenic compounds by *Pelagibacter* and possibly other bacterioplankton, coupled with arsenate resistance, results in the transfer of energy from phytoplankton to bacteria. We dub this a parasitic cycle because the release of arsenate by *Pelagibacter* in principle creates a positive-feedback loop that forces phytoplankton to continually regenerate arsenate detoxification products, producing a flow of energy to P-limited ocean regions.

## Abstract (precis)

In vast, warm regions of the oceans, phytoplankton face the problem of arsenic poisoning. Arsenate is toxic because it is chemically similar to phosphate, a scarce nutrient that phytoplankton cells need for growth. Many phytoplankton, including the commonest phytoplankton type in warm oceans, *Prochlorococcus*, detoxify arsenate by adding methyl groups. Here we show that the most abundant non-photosynthetic plankton in the oceans, SAR11 bacteria, remove the methyl groups, releasing poisonous forms of arsenic back into the water. We postulate that the methylation and demethylation of arsenic compounds creates a cycle in which the phytoplankton can never get ahead and must continually transfer energy to the SAR11 bacteria. We dub this a parasitic process and suggest that it might help explain why SAR11 bacteria are so successful, surpassing all other plankton in their numbers. Field experiments were done in the Sargasso Sea, a subtropical ocean gyre that is sometimes called an ocean desert because, throughout much of the year, there is not enough phosphorous in the water to support large blooms of phytoplankton. Ocean deserts are expanding as the oceans absorb heat and grow warmer.

## INTRODUCTION

Interactions between microbial species (*sensu lato*) that involve the exchange and transformation of small molecules have only recently become the subject of great interest in biology. Much of this interest has been driven by the proliferation of amplicon sequencing and network analysis, which have revealed potential interactions, while rarely revealing the mechanisms at work (1). In this study, we examine arsenic metabolism in the most abundant heterotrophic unicellular planktonic cell type in the ocean, SAR11 alphaproteobacteria. This work focuses on the genus *Pelagibacter* in the family *Pelagibacterales*. These are the most prevalent SAR11 types found in the ocean surface layer. A variety of metabolic strategies used by SAR11 cells to assimilate dissolved organic carbon resources have been identified in genomes and verified experimentally (2). Some *Pelagibacter* foraging strategies are unusual for heterotrophs; an example is the oxidation of volatile organic compounds, which are prime examples of leaky metabolites, compounds that leave cells by diffusion and become, in a sense, public goods (3).

Here, we report arsenic demethylation in *Pelagibacter* cells. We show that the methyl groups are oxidized to CO_2_, providing *Pelagibacter* cells with a source of energy, and we hypothesize that the release of demethylated arsenic compounds into the environment, including arsenate, a phosphate analogue, creates a cycle in which arsenate-sensitive phytoplankton cells are continuously regenerating methylated arsenic species.

Arsenate is chemically similar to phosphate, causing toxicity when enzymes substitute arsenate for phosphate in phosphorylation reactions, which are widespread across metabolism and particularly crucial for energy metabolism (4). Arsenate toxicity can be a significant problem for cells, not just in ecosystems with high arsenate concentrations, but also in systems where dissolved phosphate resources are depleted by biological productivity, resulting in P limitation and intense competition for the remaining P. Under these circumstances, dissolved arsenate/phosphate ratios become elevated, raising the rate at which arsenate is substituted for phosphate in biochemical reactions (5).

Several different cellular mechanisms have evolved to control arsenate toxicity. The most widely distributed mechanism relies mainly on two proteins, ArsC, an intracellular reductase that converts arsenate to arsenite, and ArsB, an arsenite efflux pump (6). This mechanism is found in many bacteria and some archaea and eukaryotes (7). An alternative system that is found in some fungi and both eukaryotic and prokaryotic phytoplankton involves arsenate reduction followed by the addition of one or more methyl groups by the enzyme ArsM, arsenite *S*-adenosyl methyltransferase, to form the products monomethylarsonic acid (MMA), dimethylarsinic acid (DMA), and the gas trimethylarsine oxide [TMAO(g)] (8). Methylated arsenic compounds are less toxic than arsenite. Hellweger and Lall modeled arsenate detoxification in lake phytoplankton and hypothesized that rapidly growing phytoplankton excrete arsenite when engaged in luxury phosphate uptake, explaining the observation of arsenite accumulation in the spring water column (9). In the summer, when dissolved phosphate is depleted, phytoplankton switch to a detoxification pathway that converts arsenate to arsenite and then methylates this compound to the less toxic compounds MMA and DMA, explaining the accumulation of the compounds in the water column later in the summer (9).

DMA demethylation has been demonstrated in soils, but not aquatic systems, prior to this report (9–11). Demethylation of MMA, mediated by the C-As lyase ArsI, has been demonstrated in the cyanobacterium *Nostoc* sp. strain PCC 7120 (12). In general, arsenic compound demethylation in nature and the metabolic pathways responsible have been sparsely studied.

Arsenate concentrations within the mixed layer of the North Atlantic typically range from 5 to 30 nM (13–16), The oligotrophic center of the gyre is characterized by low phosphate concentrations (<0.2 to 10 nM) (17). In the Sargasso Sea, the study site for our project, arsenate/P ratios can exceed 50 (13). Across the North Atlantic, arsenite was reported to be the most variable arsenic detoxification product, ranging in concentration from <1 to 6 nM, and had a greater negative correlation with phosphate concentrations than MMA (<0.1 to 0.4 nM) or DMA (0.6 to 8 nM) (13, 16). Seasonality in arsenic cycling in the ocean has not been studied, but the parallels between fresh and marine systems suggest that Hellweger and Lall’s model might provide a useful basis for predictions (9).

In P-depleted tropical and subtropical waters worldwide, the most prevalent phytoplankton are in the *Prochlorococcus* genus. Genes for arsenic methylation are ubiquitous in global *Prochlorococcus* populations, with additional genes for the efflux of methylated products found in populations that experience high As/P ratios, such as those in the Sargasso Sea (18). These reports suggest that phytoplankton, particularly *Prochlorococcus*, expend substantial energy converting arsenate to methylated arsenic compounds in the process of competing for trace phosphate resources in P-depleted ocean surface waters. As reported by Wurl et al. (13, 16) and others, arsenite concentrations are typically several-fold-higher than those of methylated arsenates, but rates of biological production of these compounds by detoxification metabolism, in the case of arsenite, or demethylation, in the case of methylated arsenic compounds, have not been reported (13).

Motivation for this study developed from the observation that SAR11 bacterioplankton are methylovores that utilize a tetrahydrofolate-linked pathway to oxidize a broad array of methylated and C_1_ compounds (19, 20). We hypothesized that the removal of methyl groups from MMA and DMA and the return of arsenate to the water column might form a cycle that shuttles energy from phytoplankton to SAR11. The findings we report confirm key elements of this hypothesis, notably the ability of SAR11 cells to oxidize methyl groups from DMA, and a similar activity in surface bacterioplankton communities from the western Sargasso Sea.

## RESULTS AND DISCUSSION

The metabolism that we report here, the production of cellular energy by the oxidation of methylated arsenates, has not been previously reported. We surveyed complete or nearly complete SAR11 genomes for evidence of arsenic metabolism and found only two arsenic-related genes: the LMWP family arsenate reductase (LMWPc_ArsC) and an adjacent aquaporin coding sequence located downstream on the same strand. These were present in most strains of the 1a.3 ecotype but in no strains of the 1a.1 ecotype of *Pelagibacter* (see Fig. S1 and Table S1 in the supplemental material). The 1a.1 and 1a.3 ecotypes of *Pelagibacter* have different biogeographical distributions with latitude: the 1a.1 ecotype is found in cool temperate and polar waters, whereas the 1a.3 ecotype is abundant in warm equatorial and subtropical waters (21). The catalytic mechanism of arsenate reduction by LMWPc_ArsC is similar to that of ArsC, an unrelated family of enzymes that also functions to detoxify arsenate by reduction to arsenite (22). The downstream aquaporin (AqpS) is related to a protein that has been shown to replace the ArsB gene, which encodes an active arsenite extrusion transporter, in Sinorhizobium meliloti (23). Aquaporins are passive transporters, but because of the electrostatic field across cell membranes, they are more likely to efflux negatively charged compounds of arsenic. The mammalian aquaporin AQP9 has been shown to transport MMA, as well as MMA and DMA (24).

10.1128/mBio.00246-19.1 FIG S1 Gene order in the vicinity of the LMWPc_ArsC gene. In this graph, LMWPc_ArsC is labeled “arsenate reductase ArsC.” Download FIG S1, TIF file, 0.2 MB. Copyright © 2019 Giovannoni et al. 2019 Giovannoni et al. This content is distributed under the terms of the Creative Commons Attribution 4.0 International license.

10.1128/mBio.00246-19.4 TABLE S1 Arsenic-related genes in SAR11 genomes. Manual BLASTP was used to search a database consisting of complete or nearly complete SAR11 genomes. Queries were representative genes involved in arsenic metabolism selected from the most closely related taxon available in the NCBI, e.g., alphaproteobacteria or proteobacteria. An expect of 0.01 was used as the cutoff. Hits below this threshold were manually inspected and scored as a positive if they were nearly full length. Download Table S1, DOCX file, 0.02 MB. Copyright © 2019 Giovannoni et al. 2019 Giovannoni et al. This content is distributed under the terms of the Creative Commons Attribution 4.0 International license.

**FIG 1:**
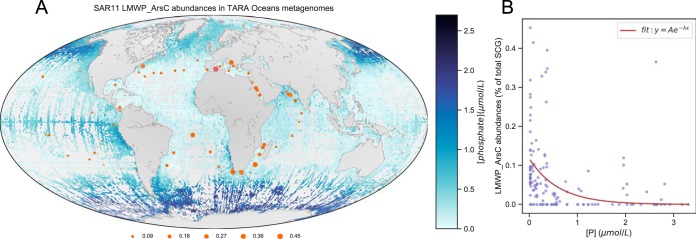
Oceanic phosphate concentrations and SAR11 LMWPc_ArsC gene relative abundances in TARA Oceans metagenomic data (expressed as percentages of the total number of SAR11 single-copy genes found per sample). (A) Global phosphate measurements from NOAA World Ocean Atlas 2013 version 2 (https://www.nodc.noaa.gov/OC5/woa13/woa13data.html) showing regions of low and high phosphate concentrations. Gray patches in the oceans indicate missing data abundances and surface phosphate concentrations measured during the TARA Oceans Expedition. (B) An inverse exponential model (inset) applied to the data yielded a *y* of 0.1176 × e^−1.653[P]^, with an *r*^2^ of 0.126. The *P* values were 3.33 × 10^−16^ for the scalar A estimate of 0.1176 and 0.006455 for the rate value estimate of −1.653. TARA Oceans phosphate measurements were obtained from Table W8 of the companion website (http://ocean-microbiome.embl.de/data/OM.CompanionTables.xlsx). A custom python script, osu_plot_TARA_arsenate_reductases_map.py (https://bitbucket.org/jimmysaw/arsenic/src/master/osu_plot_TARA_arsenate_reductases_map.py), was used to plot the distribution of SAR11 LMWPc_ArsC bacteria as a fraction of SAR11 single-copy conserved genes identified in TARA Oceans samples plotted over a world map showing global ocean surface phosphate concentrations. The same Python script also calculated the correlation of standardized LMWPc_ArsC abundances versus phosphate concentrations measured during the TARA Oceans Expedition in 2011. SCG, single-copy genes.

The distribution of ArsC in SAR11 genomes suggested that it might be most prevalent in the low-phosphate ocean regions, where stress related to arsenate toxicity is highest. To investigate the global distribution of arsenate reductase, we built a hidden Markov model (HMM) profile of LMWPc_ArsC and searched for these genes in the TARA Oceans Project metagenomes. Searches returned between 0 and 251 hits per sample, with E value thresholds lower than 1e^−3^. These hits were further filtered with Kraken (25) to exclude non-SAR11 genes. Figure 1 shows the distribution of SAR11 LMWPc_ArsC genes in TARA Oceans metagenomic data as a function of SAR11 single-copy genes.

SAR11 LMWPc_ArsC genes were variably present in SAR11 genomes but were more abundant than single-copy genes in P-limited regions of the oceans (17, 26–28). The Spearman’s rho value for this relationship was −0.56, with a *P* equal to 2.95 × e^−13^. A sample that originated from surface water (5 m) in the Mediterranean Sea (TARA_007_SRF_0.22-1.6) yielded the greatest prevalence of LMWPc_ArsC genes (0.45% of all single-copy genes). LMWPc_ArsC abundances were also high in samples collected from the Indian Ocean in the Agulhas current near the southern tip of Africa (at 0.41% and 0.39%) and along the South African coast. In the North Atlantic Ocean, the prevalence of LMWPc_ArsC genes was up to 0.36% in the South Atlantic gyre and 0.26% in the North Atlantic gyre. In contrast, LMWPc_ArsC abundances reached only 0.09% in the eastern Pacific Ocean.

Figure 1 shows a strong inverse relationship between the frequency of LMWPc_ArsC from SAR11 genomes and dissolved surface phosphate concentration in the ocean. This pattern is likely the result of positive selection for ArsC as a function of the environmental As/P ratio. Indeed, it previously has been proposed that arsenite, a product of the LMWPc_ArsC protein, can be used as a proxy for P limitation, since the As/P ratio is high in P-limited marine systems, exposing cells to greater stress from arsenate toxicity (13).

**FIG 2:**
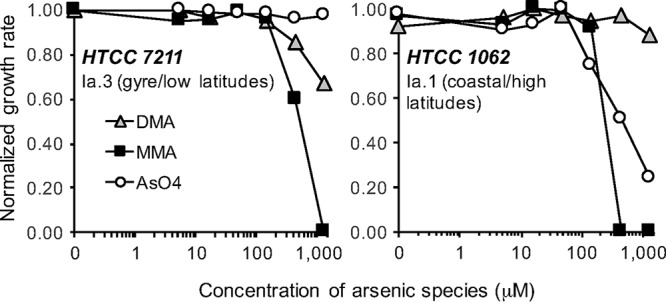
Tolerance of SAR11 cells to arsenical compounds in culture. Circles, arsenate; squares, MMA; triangles, DMA. Each data point is the slope from a log-linear regression. Growth rates for each strain were standardized to the maximum growth rate of each strain. The highest concentrations of arsenical compounds used in these experiments far exceed natural concentrations, which are typically in the nanomolar range, but As/P values in this experiment are 65:1 at the highest As concentrations (1.3 mM), which is the upper end of the range for environmentally realistic As/P ratios in the oceans.

We investigated the toxicity of arsenate, MMA, and DMA in two strains of cultured *Pelagibacter* cells: HTCC1062 and HTCC7211 (Fig. 2). These strains belong to the 1a.1 and 1a.3 ecotypes of *Pelagibacter*, respectively, and whereas HTCC1062 lacks all known genes for arsenic resistance and detoxification, HTCC7211 has LMWPc_ArsC (Table S1), as well as suites of genes that are unrelated to As but are adaptive to the low-P conditions of ocean gyres (29, 30). The *R*^2^ values for the growth curves used to estimate the growth rates shown in Fig. 2 ranged from 0.86 to 0.99 for the HTCC7211 experiments and 0.98 to 0.99 for HTCC1062. In cultures supplied with 10 µM phosphate, neither strain was inhibited by arsenate, MMA, or DMA when these compounds were added to a natural seawater medium at concentrations as high as 50 µM, but at higher concentrations, the coastal and gyre strains diverged. HTCC1062, which lacks the LMWPc_ArsC gene, was more sensitive to arsenate and MMA but less sensitive to DMA. The presence of LMWPc_ArsC in HTCC7211 is a likely explanation for its greater tolerance to arsenic. Previously, we reported that this gene is upregulated 2.3-fold in P-starved HTCC7211 cells (29, 30), but we observed no upregulation of this gene in cells exposed to DMA (Table 1). These observations suggest that LMWPc_ArsC is slightly upregulated in cells exposed to phosphate limitation. One of the differences between the coastal 1a.1 ecotype of *Pelagibacter* and the 1a.3 ecotype from warmer ecosystems is that the latter frequently lacks the glycolysis metabolic pathway (31), some versions of which are sensitive to arsenate poisoning. In the toxicity experiments shown above, cells were grown on a medium lacking glucose, so strain differences in glycolysis metabolism are an unlikely explanation for the differences in toxicity shown in Fig. 2.

**TABLE 1:** Change in gene transcription in HTCC7211 cultures exposed to 146 µM DMA

COG Database accession no.	Locus tag	Fold change	*q* value	Gene	Annotation
COG0330	NA	2.80	0.027	*hflC*	Protease modulator
COG0316	PB7211_1206	2.42	0.019	*hesB*	Iron-sulfur cluster assembly accessory protein
COG2151	NA	2.39	0.019	*paaD*	SUF system Fe-S cluster assembly protein
COG0459	PB7211_1188	2.31	0.040	*groEL*	Chaperonin
Null	PB7211_454	2.26	0.036		Oxidase
COG0520	PB7211_564	2.20	0.022	*csdB*	Cysteine desulfurase
COG1220	PB7211_449	2.11	0.019	*hslU*	ATP-dependent protease ATPase subunit
COG0171	PB7211_65	2.11	0.048	*nadE*	NAD^(+)^ synthetase
COG3221	PB7211_926	2.01	0.050	*phnD*	Phosphate/phosphite/ phosphonate ABC transporter, substrate- binding protein

To test the ability of *Pelagibacter* cells to produce energy from arsenate compounds, we assayed the ATP content of energy-starved cells after exposure to arsenate compounds (Table 2). After 16 h of starvation, ATP levels dropped dramatically in both HTCC7211 and HTCC1062 but were restored by the addition of pyruvate, a substrate that can be used by these cells for energy production. In strain HTCC7211, 10 and 30 µM DMA and 10 µM MMA addition resulted in significant increases in cellular ATP, but much lower responses were observed in HTCC1062. The evidence indicated a decreasing response with increasing MMA concentrations in HTCC7211, suggesting that, although this compound was metabolized, it had the potential to be inhibitory.

**TABLE 2:** ATP content in SAR11 strains in the presence of DMA, MMA, or pyruvate^*a*^

SAR11 strain	Ratio of ATP (SD) with:					
	Pyruvate		DMA		MMA	
	10 μM	30 μM	10 μM	30 μM	10 μM	30 μM
HTCC7211	2.77^*b*^^,^^*c*^ (0.34)	3.49^*b*^^,^^*c*^ (0.36)	2.20^*b*^ (0.38)	2.08^*b*^ (0.40)	1.74^*b*^ (0.20)	0.83 (0.08)
HTCC1062	2.76^*b*^^,^^*c*^ (0.05)	3.36^*b*^^,^^*c*^ (0.09)	1.46^*b*^ (0.03)	1.49^*b*^ (0.04)	1.00 (0.02)	1.36 (0.05)

a Data are expressed as the ratio between treatments with arsenic compounds and energy-starved controls. Cells were harvested in late exponential phase, washed once with ASW, resuspended in 2 ml ASW, and starved for 16 h. Subsamples were incubated with substrates for 28 h and then assayed for ATP content. Numbers in parentheses are SD determined by propagation of error.

b *P* value significance (pairwise *t* test) was <0.05, compared to the value for the negative control for the given strain.

c *P* value significance (pairwise *t* test) was <0.05, compared to values with DMA or MMA for the given strain.

**FIG 3:**
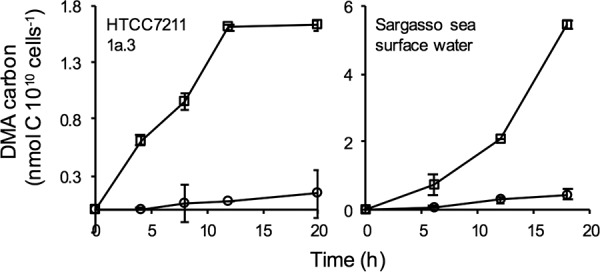
DMA utilization by HTCC7211 (left) and surface seawater collected in the Sargasso Sea (right). DMA-carbon incorporated into biomass (circles) was measured following addition of TCA and filtration. DMA-carbon oxidized to CO_2_ (squares) was measured following addition of base, Na_2_CO_3_ and BaCl_2_, and filtration. Whiskers indicate standard deviations (SD) of triplicate measurements.

The oxidation of DMA methyl groups by *Pelagibacter* cells was confirmed by synthesizing [^14^C]DMA and testing cultures for their ability to transform this compound into ^14^CO_2_ (Fig. 3). HTCC7211 oxidized [^14^C]DMA at a rate of ∼0.13 nmol 10^10^ cells^−1^ · h^−1^. Less than ∼5% of that value was assimilated into cellular biomass. These results are consistent with previous work which demonstrated that SAR11 cells are methylovores that oxidize methyl groups for energy but lack pathways for assimilating C_1_ carbon into biomass.

A concentrated suspension of Sargasso Sea surface bacterioplankton oxidized [^14^C]DMA to ^14^CO_2_ at the rate of 0.3 nmol 10^10^ cells^−1^ · h^−1^. [^14^ C]pyruvate, a common labile substrate, was oxidized at almost exactly the same rate. This corresponds to a rate of DMA oxidation of 400 pmol · liter^−1^ · day^−1^, assuming natural bacterioplankton cell densities of 5.5 × 10^8^ cells · liter^−1^. At a background DMA concentration of 3.2 nmol · liter^−1^, the average concentration reported in the central Atlantic (13), this corresponds to a potential turnover of DMA standing stocks every 8 days, which is roughly 3-fold faster than previously reported estimates (13).

**FIG 4:**
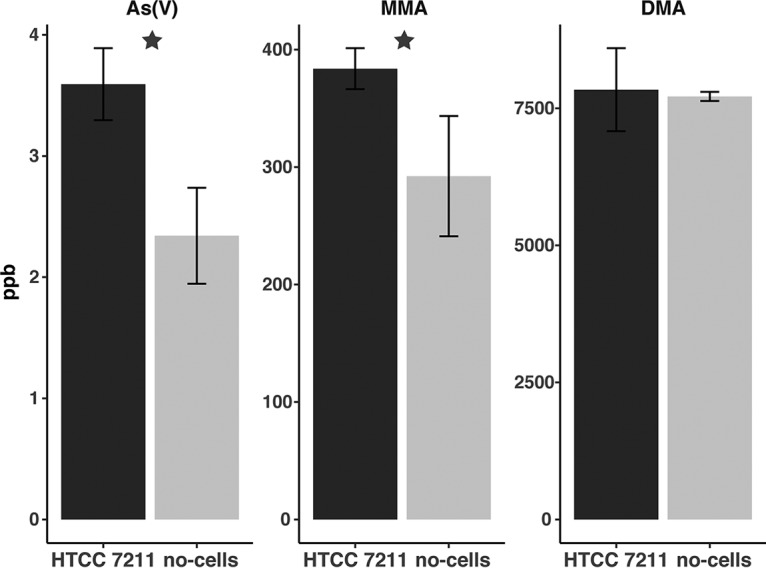
Arsenate and MMA production by HTCC7211 cultures. Mean concentrations of dissolved arsenate, MMA, and DMA from HTCC7211 cultures (dark gray; *n* = 5) and in no-cell controls (light gray; *n* = 4). Error bars are standard deviations. Stars indicate statistically significant differences (*P* value < 0.05). The starting medium was amended with 5 μM DMA.

To confirm the oxidation of DMA by *Pelagibacter* cells, we developed a method to directly measure arsenical compounds produced by SAR11 using arsenic hydride generation coupled with proton transfer reaction-time of flight (PTR-TOF) mass spectrometry (13). After a 72-h incubation in artificial seawater (ASW) medium amended with 5 µM DMA, we measured significantly higher concentrations of arsenate (*P* value = 0.003) and MMA (*P* value = 0.032) dissolved in HTCC7211 cultures than in the no-cell positive control (Fig. 4). No significant difference in DMA concentration was observed between treatments, but that was expected, since an excess (5 μM) was added. No arsenite was detected in any treatment. The mean dissolved arsenate concentrations in the HTCC7211 culture and no-cell positive control were 3.59 ± 0.30 ppb and 2.34 ± 0.40 ppb, respectively, while the mean dissolved MMA concentrations were 383.7 ± 17.4 ppb and 292.3 ± 51.2 ppb, respectively. These data demonstrate the production of arsenate and MMA from DMA by HTCC7211. Further, they suggest a multistep oxidation process for DMA, where methyl groups are oxidized in a stepwise fashion.

**FIG 5:**
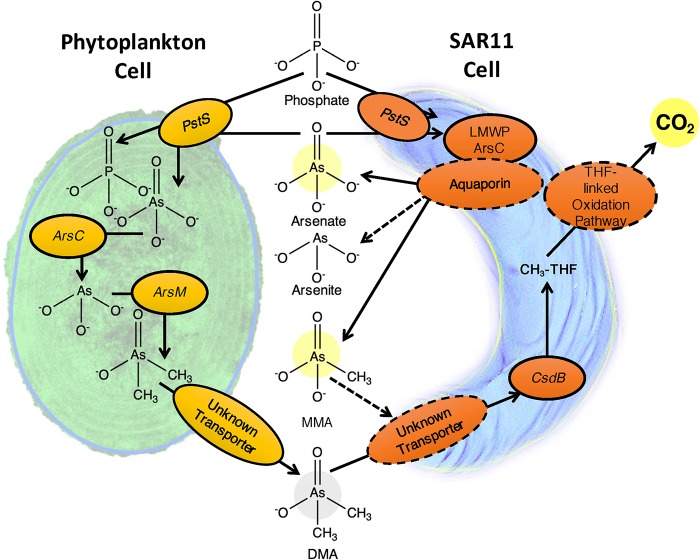
Proposed parasitic arsenic cycle. Solid arrows represent processes and enzymes that have been experimentally proven in this study or elsewhere, while dashed lines represent proposed connections and enzymes that have not yet been experimentally determined. Phytoplankton, represented by the *Prochlorococcus* cell on the left, detoxify arsenate by methylation, resulting in accumulation of methylated arsenic compounds in the environment. Methylated arsenic compounds, represented here by DMA and MMA, are demethylated by *Pelagibacter*, resulting in the release of inorganic arsenic or MMA (single demethylation of DMA) into the environment. In this study, we have observed the demethylation of DMA (gray circle) and the resultant production of MMA, arsenate, and CO_2_ (yellow circles). We postulate that LMWPc_ArsC, an arsenate reductase, and the associated aquaporin constitute an arsenic toxicity resistance module. Many parts of this scheme are speculative, for example, the role of CsdB. The tetrahydrofolate-linked C_1_ oxidation pathway in *Pelagibacter* has been demonstrated to produce energy via oxidation of methyl groups but does not supply net organic carbon for growth; hence, this proposed cycle is a form of energy parasitism.

Affymetrix microarrays were used to measure the transcriptional response of *Pelagibacter* HTCC7211 to the addition of DMA (Table 1). None of the genes for which transcription changed significantly in response to DMA addition could be clearly linked to arsenic metabolism, but several of the upregulated genes offer potential insights into the metabolism of methylated arsenic compounds by *Pelagibacter* cells. The gene *csdB*, which is annotated as cysteine desulfurase, encodes a C-terminal domain that is homologous to the shorter As-C lyase *arsI* from *Nostoc* sp. PCC 7120 (Fig. S3). This protein has been demonstrated to demethylate MMA (12). *csdB* was found in nearly all SAR11 genomes (Table S2). DMA addition also stimulated a 2-fold upregulation of *phnD*, a transporter for phosphonate compounds, which include analogs of MMA (29). These insights were useful for building the proposed scheme for arsenic compound metabolism in *Pelagibacter* (Fig. 5).

10.1128/mBio.00246-19.2 FIG S2 Protein sequence alignment of LMWPc_ArsC proteins from HTCC7211 (number 1), LMWPc_ArsC proteins from *Synechocystis* sp. strain PCC6803 (number 2), Staphylococcus aureus (number 3), and Bacillus subtilis (number 4). Positions 10 and 85 in the consensus sequence are conserved catalytic cysteine residues identified in reference 1, which demonstrates the mechanism of arsenate reduction in these proteins. The figure was created with Geneious version 11.1. Download FIG S2, TIF file, 0.2 MB. Copyright © 2019 Giovannoni et al. 2019 Giovannoni et al. This content is distributed under the terms of the Creative Commons Attribution 4.0 International license.

10.1128/mBio.00246-19.3 FIG S3 Protein sequence alignment of the proteins CsdB (annotated as cysteine desulfurase) from HTCC7211 and ArsI from *Nostoc.* The C-terminal 276 amino acids of CsdB are homologous to the *Nostoc arsI* gene, which has been experimentally tested *in vitro* and shown to demethylate MMA, producing arsinite as a product (12). Download FIG S3, EPS file, 0.8 MB. Copyright © 2019 Giovannoni et al. 2019 Giovannoni et al. This content is distributed under the terms of the Creative Commons Attribution 4.0 International license.

10.1128/mBio.00246-19.5 TABLE S2 Distribution of arsenic-related genes among complete or nearly complete SAR11 genomes from isolates. Download Table S2, DOCX file, 0.02 MB. Copyright © 2019 Giovannoni et al. 2019 Giovannoni et al. This content is distributed under the terms of the Creative Commons Attribution 4.0 International license.

Our findings show that energy, in the form of methyl carbon, is harvested by *Pelagibacter* cells from arsenic detoxification products formed by phytoplankton. From these data, we postulate the existence of a parasitic arsenic cycle that is sensitive to environmental As/P ratios and most active in oligotrophic, P-depleted ocean regions. Perhaps not coincidently, it is in these ocean regions that *Pelagibacter* strains related to those that we studied are most dominant, sometimes reaching 40% of ocean surface plankton communities (32). We apply the term “parasitic” to this cycle because it draws energy from phytoplankton, to their detriment, and because, in principle, the arsenical compounds released by *Pelagibacter* become available again for competitive uptake by phytoplankton phosphate transporters, thus requiring further rounds of detoxification. Our data suggest that in the western Sargasso Sea, the entire pool of methylated arsenic compounds might turn over by this mechanism every 8 days.

In the conceptual model of the parasitic arsenic cycle that we propose (Fig. 5), the bacterial heterotrophs that transform methylated arsenic compounds to toxic products are not parasites in the usual sense, which involves evolution to directly interact with hosts. Rather, the origin of this cycle was likely opportunism, in the sense that methylated arsenic compounds released into the environment became substrates that could be exploited, much as other products of phototrophic metabolism are potential resources for heterotrophs. The difference in the case of arsenic is the feedback loop created by the release of toxic arsenic products, particularly arsenate, when the methylated arsenic resources are exploited. This feedback loop completes a bidirectional interaction that is likely entirely to the benefit of *Pelagibacter* and to the detriment of phytoplankton. Additionally, the unusual metabolism of *Pelagibacter*, which lacks enzymes for transforming C_1_ compounds into biomass, requires that this process transfers energy mainly from methyl group oxidation, but not carbon for biomass, from phototrophs to heterotrophic cells. Thus, the parasitic arsenic cycle as we postulate it is an indirect form of energy parasitism.

The energetics of the model that we propose in principle might produce substantial energy income for the “parasite.” The tetrahydrofolate (THF)-dependent oxidative pathway produces two reduced nucleotide cofactors for each methyl group oxidized, potentially yielding four reduced nucleotide cofactors for each DMA molecule oxidized. Aquaporin family proteins are passive ion channels that transport negatively charged arsenic compounds to the cell’s exterior, dissipating some potential energy. By the accounting above, the oxidation of a single DMA with arsenate produced as the product would yield about 10 ATP, not accounting for transport costs. These predictions are in accord with the observations that we report of elevated ATP levels when energy-starved cells are exposed to DMA.

It is uncertain how many taxa participate in the parasitic arsenic cycle or to what degree. However, the most abundant phytoplankton in the oceans, *Prochlorococcus*, have been shown to possess genes for detoxifying arsenic by methylation, and here we show that *Prochlorococcus*’ heterotrophic counterpart, *Pelagibacter*, completes a parasitic cycle of arsenate regeneration. One of the challenges in deciphering this cycle and understanding its influence on planktonic processes is the paucity of information about the biochemical steps involved. Only recently was the demethylation of MMA shown to be mediated by the C-As lyase *arsI* in the cyanobacterium *Nostoc* sp. PCC 7120 (12), but the protein was not tested for its ability to utilize DMA as a substrate. Here, we report a much larger protein that includes the domain encoded by the *Nostoc arsI* gene, as upregulated by DMA, making it a potential candidate for this function. Some proteins encoded by the *Pelagibacter* genome that are not annotated as having arsenic-related functions have catalytic mechanisms which suggest that they might play a role in arsenic metabolism. For example, previously, we described a C-P lyase in HTCC7211 that is induced by P starvation and cleaves the C-P bonds of phosphonates, including methyl phosphonate, to release methane (29, 30). Because of the chemical similarity of As to P, it is possible that a promiscuous activity of this enzyme might cleave methyl groups from arsenic compounds when these cells are induced by P limitation.

We recently estimated that between 6 and 37% of ocean gross primary production is oxidized by *Pelagibacter* cells (33). The evidence of arsenic cycling that we report here may help to explain how *Pelagibacter* cells achieve such a high productivity in the most oligotrophic regions of the oceans, which, we note, are expanding as the oceans warm.

## MATERIALS AND METHODS

### Bioinformatics analysis.

A total of 231 representative amino acid sequences belonging to the low-molecular-weight phosphatase arsenic reductase (LMWPc_ArsC) protein family were downloaded from the conserved domain database (https://www.ncbi.nlm.nih.gov/Structure/cdd/cddsrv.cgi?uid=cd16345), and the sequences were aligned using MAFFT-linsi multiple-sequence alignment tool (34). Aligned sequences were trimmed to remove poorly conserved regions using trimAl (35), with the following parameter: -gt 0.5. Trimmed alignment was then used to build a hidden Markov model (HMM) of LMWPc_ArsC using HMMER version 3.1b1 (36), and the HMM built was used to search for putative arsenate reductases in the predicted amino acid sequences from 243 TARA Oceans Project metagenomic samples (37).

All coding DNA sequences of TARA Oceans proteins matching the LMWPc_ArsC HMM profile were classified using Kraken2 (25) to identify sequences belonging to SAR11 genomes. Twenty-one pure culture isolate genomes and 29 randomly selected metagenome-assembled genomes (MAGs) were downloaded from two publicly available databases (NCBI and JGI), and coding DNA sequences from the TARA Oceans metagenome were searched against these genomes using Kraken2 with default parameters. A curated set of HMM profiles of 109 single-copy bacterial marker genes was obtained from the following website: https://bitbucket.org/lionelguy/phyloskeleton/src/master/share/Bact109.hmm (38), and HMM profiles were searched against the amino acid sequences of SAR11 reads identified in the TARA Oceans metagenomes. This step was necessary to identify SAR11 single-copy marker genes present in TARA Oceans metagenomes, and the gene counts were used to standardize SAR11 arsenate reductase gene abundances in each TARA Oceans metagenome sample. All the TARA Oceans metagenomic reads matching LMWPc_ArsC were also classified using Kraken2 to identify arsenate reductases that are specific to the SAR11 clade bacteria.

### Culture conditions.

For measuring arsenate compound toxicity, the transcriptional response to DMA, and [^14^C]dimethyl arsenate oxidation activity, cultures were grown in a sterile seawater medium, low-nutrient heterotrophic medium (LNHM), as described elsewhere (39). For measuring transcriptional response and [^14^C]dimethyl arsenate oxidation activity, the medium was amended with 1 μM FeCl_3_, 1 mM NH_4_Cl, 100 μM KH_2_PO_4_, 100 μM pyruvate, 50 μM glycine, and 50 μM methionine; for measuring arsenic compound toxicity, the KH_2_PO_4_ concentration in the seawater medium was reduced to 10 μM KH_2_PO_4_. Oregon coastal seawater was collected with Niskin bottles from station NH-5 ca. 9 miles offshore at 44°39.1′N. Cells were harvested by centrifugation on a Beckman J2-21 centrifuge at 10°C at 30,000 × g for 90 min, followed by resuspension in unamended ASW (no added organics or phosphorous), a second centrifugation step in a Beckman-Coulter ultracentrifuge at 12°C at 48,000 × g for 60 min, and a final resuspension in ASW. For the direct measurements of DMA demethylation product production by PTR-MS, described below, the cells were grown on artificial seawater medium (40). For all experiments, cells were counted by staining them with the DNA stain SYBR green I (Invitrogen) for 1 h, followed by enumeration with a flow cytometer (Guava Technologies) (41).

### [^14^C]dimethyl arsenate synthesis.

Methyldiiodoarsine was prepared as described by Millar et al. (42) and reacted with [^14^C]iodomethane, as described by Jagadish et al. (43), to produce [^14^C]dimethylarsenate. For this synthesis, 5 mCi [^14^C]iodomethane (specific activity, 54 mCi/mmol in 50 µl methanol) was purchased from American Radiolabeled Chemicals and reacted with 92.6 µM methyldiiodoarsine in 100 µl methanol (MeOH) in a 1-dram Wheaton v-vial. The reaction was quenched by the addition of 37 µl of 10 N sodium hydroxide. The products were then purified by acetone precipitation, producing [^14^C]DMA with a specific activity of 3 mCi/mM.

### [^14^C]dimethyl arsenate uptake and oxidation assays in culture.

*Pelagibacter* HTCC7211 cells were harvested in log phase by centrifugation and resuspended in fresh medium to about 5 × 10^7^ cells ml^−1^. [^14^C]DMA (100 nM) was added to both live and killed culture samples; negative controls (killed) were incubated in 10% formalin for 1 h before the addition of the isotope to the sample. Inoculated cultures (20 ml) were aliquoted into 160-ml glass vials, which were then sealed with Teflon-lined stoppers. Samples were incubated in the dark at 17°C. At each time point, reagents were added to cultures using syringes inserted through stoppers.

For ^14^C incorporation, 100% (wt/vol) cold trichloroacetic acid (TCA) was added to a final concentration of 10%. To precipitate ^14^CO_2_, 0.2 ml 1 M NaOH, 0.5 ml 0.1 M Na_2_CO_3_, and 1 ml 1 M BaCl_2_ were added to form Ba^14^CO_3_ and Ba(OH)_2_ (44). All samples were collected by filtration after incubation at 4°C for 12 h. Precipitates were collected on 0.2-μm nitrocellulose Global Soil Wetness Project (GSWP) filters (Millipore, Billerica, MA), washed three times with 3 ml of ice-cold 5% (wt/vol) TCA (^14^C incorporation) or 3 ml of ice-cold fresh SAR11 growth medium (^14^C oxidation), and transferred to scintillation vials. Filters were transferred to vials containing 5 ml Ultima Gold XR scintillation fluid (Perkin-Elmer), shaken vigorously, sonicated (to disrupt caked precipitate in ^14^C oxidation samples), and kept in the dark overnight before organisms were counted (Beckman LS-6500 liquid scintillation counter).

### [^14^C]dimethyl arsenate uptake and oxidation assays in the western Sargasso Sea.

Seawater samples of 230 liters were collected from a 20-m depth with Niskin bottles from Hydrostation S in the western Sargasso Sea on 1 and 2 July 2015 and concentrated by tangential-flow filtration for measurements of live cell activity with ^14^C-labeled substrates as described previously (19). For measurements of pyruvate incorporation and oxidation (1 July 2015), the bacterial cell concentration was 1.42 × 10^7^ cells/ml. For measurements of dimethyl arsenate incorporation and oxidation (2 July 2015), the bacterial cell concentration was 4.79 × 10^7^ cells/ml.

### Transcriptional response to dimethyl arsenate.

HTCC7211 cells were grown on LNHM medium, as described above, to ca. 10^8^ cells/ml. Replicate cultures (*n* = 3) received 145.9 μM dimethyl arsenate (DMA), while replicate control cultures (*n* = 3) received no DMA amendment. Culture growth was tracked daily by staining cells with SYBR green and counting them on a Guava easyCyte flow cytometer (41). Samples used in microarray experiments were collected during log phase. Forty microliters of culture from each biological replicate was harvested by centrifugation, resuspended in 700 μl of RNAprotect bacterial reagent (Qiagen catalog number 76506), and allowed to sit for 15 min. Cells were centrifuged again (30 min at 40,000 × g, 10°C). After decanting of the supernatant, samples were stored frozen at −80°C prior to extraction.mRNA was prepared by resuspending cell pellets in 100 µl TE (1 mM Tris, 1 mM EDTA; pH 8) containing 40 µg lysozyme and incubated at room temperature for 5 min. Total RNA was extracted using RNeasy MinElute cleanup kits (Qiagen catalog number 74204) according to the manufacturer's instructions, with the exception of an additional wash step with 700 µl buffer RW1 (Qiagen catalog number 1053394) immediately prior to the prescribed washes with buffer RPE. Eluted RNA was amplified and biotin labeled using the MessageAmp-II bacterial RNA amplification kit (Ambion catalog number AM1790) and biotin-11-UTP (Ambion) according to the manufacturer's instructions. Resulting amplified and labeled RNA (aRNA) was screened for length and quality using a Bioanalyzer 2100 (Agilent) and quantified utilizing a NanoDrop 1000 spectrophotometer (Thermo Fisher Scientific).

Protocols for microarray analysis were as described previously by Smith et al. (45). Five micrograms of biotinylated aRNA from triplicate samples was fractionated and then hybridized (45°C) overnight to custom “*Candidatus* Pelagibacter ubique” Affymetrix GeneChip arrays that contained probes for HTCC1002, HTCC1062, and HTCC7211 using Affymetrix GeneChip Fluidics Station 450 and an Affymetrix GeneChip hybridization oven, model 640. Arrays were then washed by following the manufacturer's instructions, and the resulting images were analyzed using an Affymetrix GeneChip scanner 3000. Microarray images were processed and raw data were extracted with Affymetrix GeneChip Command Console (AGCC) software. We controlled the false-discovery rate using the method of Storey and Tibshirani (46); we assigned a *q* value threshold of 0.05, which yielded 9 significant tests (Table 1) and an effective false-discovery rate of 0.45.

Differences between treatments were included in the table if both the adjusted *q* value was ≤0.05 and the gene was differentially regulated by ≥2.0-fold. *q* values were estimated using the *q* value package in the R environment.

### Chemical determination of arsenical production by HTCC7211.

Cells were harvested and washed in ASW, as described above. The cell pellets were resuspended in unamended ASW with 1 µM KH_2_PO_4_, 1× vitamin mix, and 5 µM dimethyl arsenate added. The final cell density in the resuspension was 8 × 10^8^ cells/ml. Cells were incubated in the DMA medium for 72 h under the original incubation conditions before the cells were removed via filtration on 0.1-µm filters. Negative (no DMA added) and positive (5 µM DMA added) no-cell ASW controls were included. The filtrate from all samples and controls was then analyzed for arsenical production.

Chemical determination of arsenical compounds was performed using proton transfer reaction time of flight (PTR-TOF) mass spectrometry coupled with established hydride generation methods (13, 47). Two separate analyses, one that determined arsenite and another that determined MMA, DMA, and the sum of arsenate and arsenite, were conducted. Arsenate concentrations were determined by subtracting the arsenite concentration from the arsenate plus arsenite concentration. For both methods, 5 ml of filtered sample was diluted to 30 ml with Nanopure water, and the sample was purged continuously using nitrogen gas at a flow rate of 50 ml/min. Arsenite was measured by adding 1 ml 2.5 M Tris HCl followed by hydride generation using 1 ml 4% NaBH_4_. Arsenate plus arsenite, MMA, and DMA were measured by addition of 1.5 ml concentrated HCl, followed by reduction using 2 ml 1 M KI and finally hydride generation using 3 ml 4% NaBH_4_. The total reaction time for both methods was 15 min. For both methods, concentrations of the evolved hydride gasses (arsine, monomethyl arsane, and dimethyl arsane) were continuously measured using an Ionicon PTR-TOF 1000 mass spectrometer (Ionicon Analytic). The resulting data were processed using the PTR-Viewer software package, and peak integration and statistical analysis were performed in the R software environment. Nanopure water blanks were used to establish the baseline signal for all compounds, and the mean baseline signal was subtracted from all data. Standards of all compounds were used to validate the method and to establish sensitivity and precision. The detection limit was 800 pM. This method is capable of determining highly exact mass values and relative concentrations of arsenical compounds. However, we are unable to determine the exact concentrations of the target compounds in the sample. The parts per billion (ppb) units that we report are the concentrations detected in the sparging gas entering the PTR-TOF mass spectrometer, not the concentrations of the compounds in the cultures themselves.
