# Supplementary figures and images for: A Parasitic Arsenic Cycle That Shuttles Energy from Phytoplankton to Heterotrophic Bacterioplankton
Source: mBio. 2019 Mar 19;10(2):e00246-19. doi: 10.1128/mBio.00246-19 (PMC6426599; doi:10.1128/mBio.00246-19)

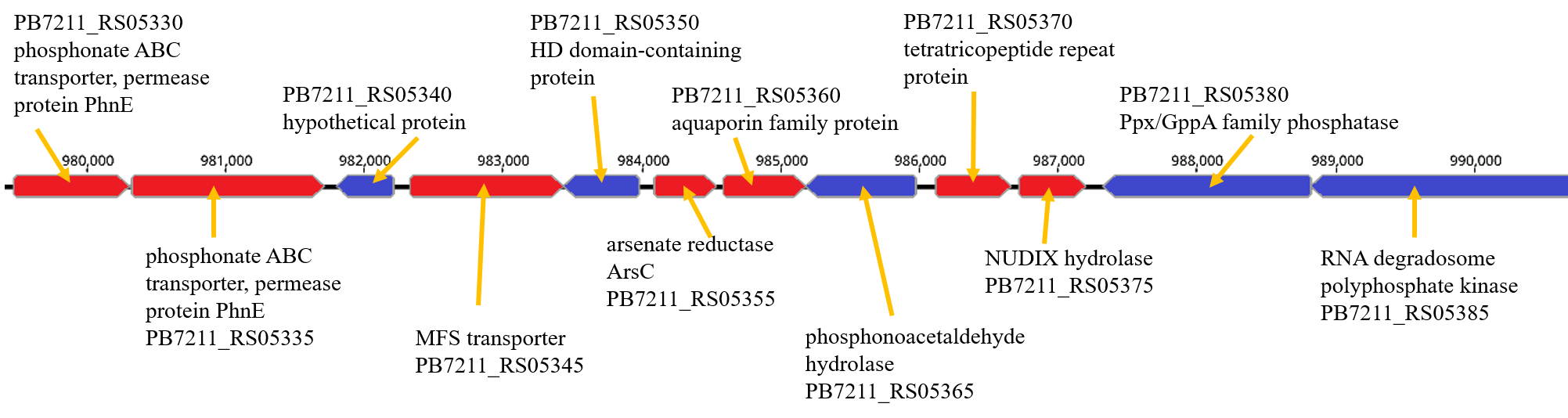

Supplement: FIG S1 [file mBio.00246-19-sf001.tif]

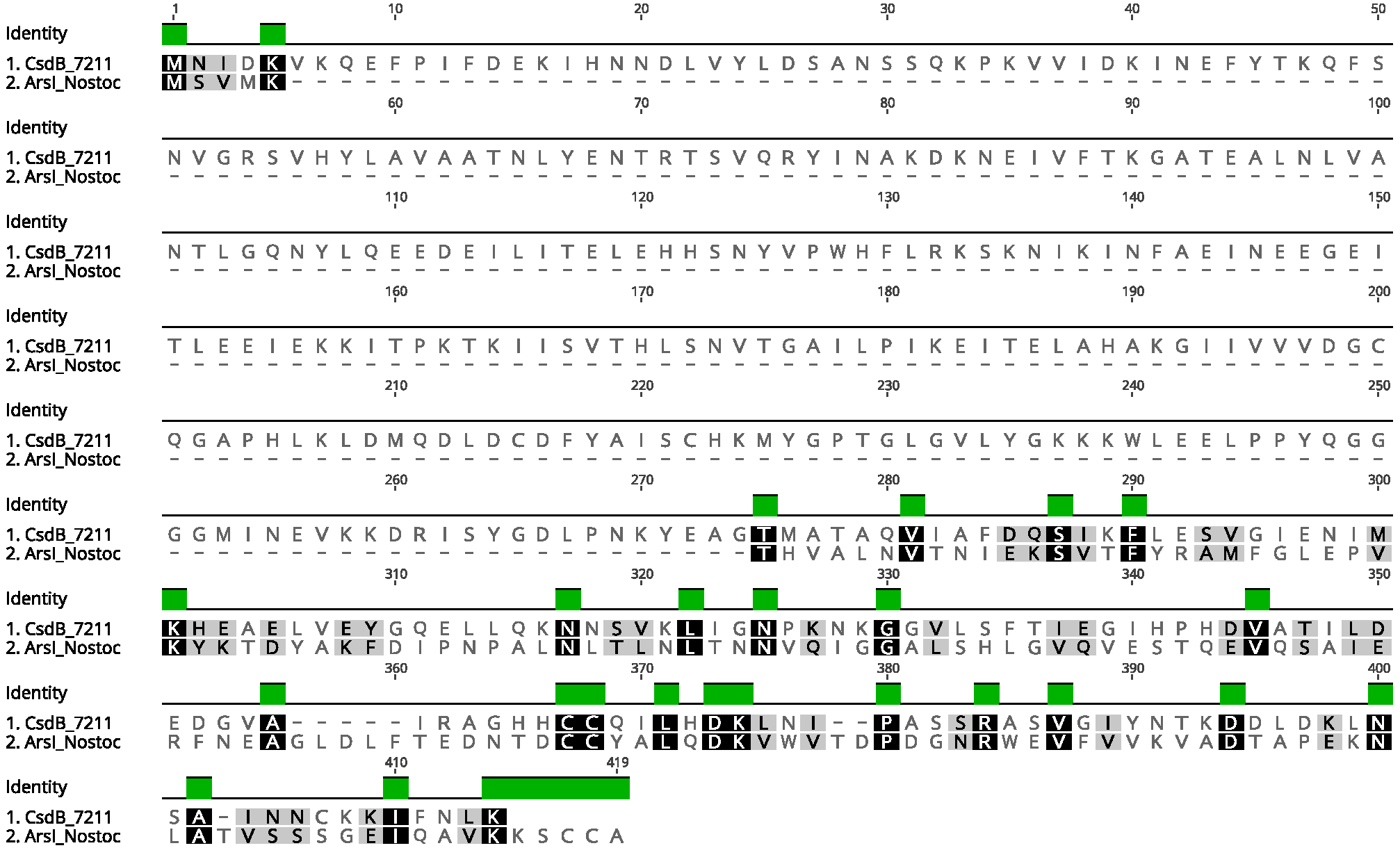

Supplement: FIG S2 [file mBio.00246-19-sf002.tif]
