# Supplementary material for: A Parasitic Arsenic Cycle That Shuttles Energy from Phytoplankton to Heterotrophic Bacterioplankton
Source: mBio. 2019 Mar 19;10(2):e00246-19. doi: 10.1128/mBio.00246-19 (PMC6426599; doi:10.1128/mBio.00246-19)
Supplement: TABLE S1 [file mBio.00246-19-st001.docx]

| **Table S1.** Arsenic-related genes in SAR11 genomes. Manual BLASTP was used to search a database consisting of complete or nearly complete SAR11 genomes. Queries were representative genes involved in arsenic metabolism selected from the most closely related taxon available in NCBI, e.g., Alphaproteobacteria or Proteobacteria. An expect of 0.01 was used as the cutoff. Hits below this threshold were manually inspected and scored as a positive if they were nearly full-length. | | | | | |  |  |  |  |  |  |  |  |  |  |
| --- | --- | --- | --- | --- | --- | --- | --- | --- | --- | --- | --- | --- | --- | --- | --- |
| **Gene Name** | **Gene Function** | **Query** | | **Outcome** | |  |  |  |  |  |  |  |  |  |  |
| ArsA | arsenical pump-driving ATPase | *Enterococcus malodoratus* | | N.D. | |  | | |  |  |  |  |  |  |  |
| ArsB | Transmembrane carrier pump | *Shewanella* sp. | | N.D. | |  | | |  |  |  |  |  |  |  |
| ArsC | arsenate reductase | *Ectothiorhodospira sp. BSL-9* | | LMWcArsC detected in most Ia.3 strains | | | | | | |  |  |  |  |  |
| ArsD | Transcriptional repressor | *E. coli* K12 | | N.D. | |  | | |  |  |  |  |  |  |  |
| ArrA | respiratory arsenate reductase | ArrA [*Shewanella* sp. ANA-3] | | hits to formate dehydrogenase, alpha subunit | | | | | | |  |  |  |  |  |
| ArrB | respiratory arsenate reductase | ArrB [*Shewanella* sp. ANA-3] | | hit to fdxA | |  | | |  |  |  |  |  |  |  |
| AioXSR | signal transduction | null | | All two-component systems in these cells have other assigned  functions | | | | | | | | |  |  |  |
| AioA | arsenate reductase (azurin) large subunit | *Herminiimonas arsenicoxydans* | | hits to fdhA | |  | | |  |  |  |  |  |  |  |
| AioB | arsenate reductase (azurin) small subunit | *Herminiimonas arsenicoxydans* | | N.D. | |  | | |  |  |  |  |  |  |  |
| ArxC | arsenite oxidase, phototrophic bacteria | not in NCBI | | N.D. | |  | | |  |  |  |  |  |  |  |
| ArsH | arsenical resistance protein | *Caulobacter segnis* | | N.D. | |  | | |  |  |  |  |  |  |  |
| ArsI | As-C lyase | *Nostoc* nsArsI | | C-terminus hits to CsdB in all SAR11 genomes | |  | | |  |  |  |  |  |  |  |
| ArsJ | MFS-type efflux pump ArsJ specific for 1-arseno-3-phosphoglycerate | hydrothermal vent metagenome | | N.D. | |  | | |  |  |  |  |  |  |  |
| ArsK | efflux transporter (As (III), roxarsone (III), methylarsenite) | not in NCBI | | null | |  | | |  |  |  |  |  |  |  |
| ArsM | arsenite methyltransferase | *Solemya velum* gill symbiont | | most strains have a ubiquinone/menaquinone biosynthesis  methyltransferase (UbiE) that is homologous to ArsM | | | |  |  |  |  |  |  |  |  |
| ArsN | no function | not in NCBI | | null | |  | | |  |  |  |  |  |  |  |
| ArsO | flavin binding monooxygenase | *Streptomyces* | | hits to TMAO oxidase | | | | |  |  |  |  |  |  |  |
| ArsP | efflux pump | *Shewenella* | | hits to TMA oxidase | | | | |  |  |  |  |  |  |  |
| ArsT | thioredoxin reductase: | *Shewenella* | | most strains have HQ hits to trxB, thioredoxin reductase | | | | | | | |  |  |  |  |
| ArsR | DNA-binding transcriptional repressor | *E. coli* str. K-12 | | N.D. | |  | | |  |  |  |  |  |  |  |
| AoxB | formate dehydrogenase | *Sinorhizobium* sp. M14 | | hits to fdhA | |  |  |  |  |  |  |  |  |  |  |
| N.D.: Not Detected | | |  | |  | |  | |  |  |  |  |  |  |  |
