# Supplementary material for: A Parasitic Arsenic Cycle That Shuttles Energy from Phytoplankton to Heterotrophic Bacterioplankton
Source: mBio. 2019 Mar 19;10(2):e00246-19. doi: 10.1128/mBio.00246-19 (PMC6426599; doi:10.1128/mBio.00246-19)
Supplement: TABLE S2 [file mBio.00246-19-st002.docx]

**Table S2** - Distribution of arsenic-related genes among complete or nearly complete SAR11 genomes from isolates.

| Strains | Ecotype | LMWP_ArsC | Aquaglyceroporin | CsdB | Origin |
| --- | --- | --- | --- | --- | --- |
| HTCC1002 | Ia.1 | 0 | 0 | 1 | Oregon Coast |
| HTCC1013 | Ia.1 | 0 | 0 | 1 | Oregon Coast |
| HTCC1016 | Ia.1 | 0 | 0 | 1 | Oregon Coast |
| HTCC1040 | Ia.1 | 0 | 0 | 1 | Oregon Coast |
| HTCC1062 | Ia.1 | 0 | 0 | 1 | Oregon Coast |
| HTCC9565 | Ia.1 | 0 | 0 | 1 | Oregon Coast |
| HIMB083 | Ia.3 | 1 | 1 | 1 | Hawaii |
| HIMB122 | Ia.3 | 1 | 1 | 1 | Hawaii |
| HIMB1321 | Ia.3 | 1 | 1 | 1 | Hawaii |
| HIMB140 | Ia.3 | 1 | 1 | 1 | Hawaii |
| HIMB4 | Ia.3 | 0 | 0 | 1 | Hawaii |
| HIMB5 | Ia.3 | 0 | 0 | 1 | Hawaii |
| HTCC7211 | Ia.3 | 2* | 1 | 1 | Sargasso Sea |
| HTCC7214 | Ia.3 | 1 | 1 | 1 | Sargasso Sea |
| HTCC7217 | Ia.3 | 1 | 1 | 1 | Sargasso Sea |
| HTCC8051 | Ia.3 | 0 | 0 | 1 | Oregon Coast |
| HTCC9022 | Ia.3 | 0 | 0 | 1 | Oregon Coast |
| HIMB058 | II | 0 | 0 | 1 | Hawaii |
| HIMB114 | III | 1 | 0 | 1 | Hawaii |
| IMCC9063 | III | 0 | 0 | 1 | Arctic Ocean |
| HIMB59 | V | 1 | 0 | 1 | Hawaii |

* Indicates that the second LMWP_ArsC copy in HTCC7211 is truncated and likely non-functional. SAR11 strains encoding at least a copy of functional LMWP_ArsC are shown in bold.
